# Real-World Impact of Insurance Changes on Long-acting Cabotegravir Plus Rilpivirine Delivery

**DOI:** 10.1093/ofid/ofag211

**Published:** 2026-04-03

**Authors:** Jennifer M Davis, Jennifer O’Neill, Elizabeth Lyden, Sara H Bares

**Affiliations:** Division of Infectious Diseases, University of Nebraska Medical Center, Omaha, Nebraska, USA; Division of Infectious Diseases, University of Nebraska Medical Center, Omaha, Nebraska, USA; College of Public Health, University of Nebraska Medical Center, Omaha, Nebraska, USA; Division of Infectious Diseases, University of Nebraska Medical Center, Omaha, Nebraska, USA

**Keywords:** HIV treatment, insurance changes, long-acting antiretrovirals, long-acting cabotegravir plus rilpivirine

## Abstract

Long-acting injectable cabotegravir plus rilpivirine offers an alternative to daily oral antiretroviral therapy, but insurance-related barriers can disrupt implementation. We examined the frequency and impact of insurance changes in a United States cohort and found that nearly half of patients experienced insurance changes, which could lead to delayed injections.

Long-acting cabotegravir plus rilpivirine (LA CAB/RPV) was approved by the US Food and Drug Administration [1] in 2021 for use as a switch strategy for people with HIV (PWH) who are virologically suppressed on oral antiretroviral therapy (ART) without a history of treatment failure. Since its approval, many clinics across the United States have developed long-acting injectable (LAI) ART programs and have identified several barriers and facilitators to the successful implementation of such programs [2–5]. Early reports emphasize the importance of deliberate patient selection, structured workflows, and coordination across pharmacy, nursing, and case management teams [6, 7]. Recognizing the complexity of implementation, the HIV Medical Association and the Infectious Diseases Society of America recently issued policy recommendations highlighting insurance processes as a key barrier to equitable access to LAI therapies [8]. Because of their high acquisition cost, LAI ART medications often require prior authorization (PA) or occupy higher formulary tiers across many public and private insurance plans, creating additional administrative barriers to timely access. Real-world studies have further identified insurance-related barriers, including PA delays, lapses in coverage, and variability in AIDS Drug Assistance Program (ADAP) formularies, as common challenges that can disrupt timely dosing and threaten program stability [4, 9, 10].

However, no studies to date have systemically evaluated the frequency and consequences of insurance changes after initiation of LAI ART, leaving an important gap in understanding how ongoing coverage instability affects continuity of care. To address this gap, we quantified the frequency of insurance changes among patients receiving LAI ART at our US Midwestern Ryan White–funded HIV clinic and examined their association with delayed injections or use of oral bridging.

## METHODS

### Study Design, Setting, and Participants

We conducted a single-center retrospective review of PWH who received 1 or more LA CAB/RPV injections at the University of Nebraska Medical Center's (UNMC’s) Specialty Care Center (SCC; a Ryan White–funded HIV clinic) between May 4, 2022 (first commercial injection at our site), and June 30, 2025. The study was approved by the Institutional Review Board at UNMC with a waiver of informed consent (Protocol #0015–25-EP). Demographics and clinical characteristics were abstracted, including insurance type, as well as the number and timing of insurance changes while on therapy. Delayed injections and/or use of an oral bridge were documented, along with reasons and outcomes. A delay in dose was defined as any injection given beyond the +7-day window or the need for an oral bridge due to an anticipated injection delay.

### Statistical Analysis

Patient characteristics were summarized using descriptive statistics. The median days between insurance changes were compared between payors. Each insurance change was considered an independent event. For patients with no insurance changes, time to change was calculated from date of initial authorization to end of study (June 30, 2025). For patients with ≥1 change, the payor was the insurance status before the change. Time to change was calculated from date of previous insurance authorization to date of subsequent authorization. The Kruskal Wallis test was used to compare median days to insurance change between the 3 payor categories. When the overall *P* value was statistically significant, pairwise comparisons between categories were adjusted using the Dwass, Steel, Critchlow-Fligner multiple comparison method. All analyses were conducted in SAS, version 9.4; a *P* value <.05 was considered statistically significant.

## RESULTS

### Baseline Characteristics

A total of 127 patients were included in the analysis, with a median age (range) of 43 (19–77) years. Most patients were cisgender men (104; 81.9%), with 19 (15%) cisgender women, 2 (1.6%) transgender women, and 2 (1.6%) nonbinary (Table 1). Most patients were White (98; 77.2%), with 25 (19.7%) Black and 20 (15.7%) Hispanic/Latinx.

**Table 1.** Patient Characteristics (n = 127)

Patient Characteristics	
Age, median (range)	43.00 (19.00–77.00)
**Gender, No. (%)**	**…**
Female	19 (15.0)
Male	104 (81.9)
Nonbinary	2 (1.6)
Transgender female	2 (1.6)
**Race, No. (%)**	**…**
American Indian or Alaksa Native	1 (0.8)
Asian	3 (2.4)
Black or African American	25 (19.7)
White or Caucasian	98 (77.2)
**Ethnicity, No. (%)**	**…**
Hispanic	20 (15.7)
Not Hispanic	107 (84.3)
**Insurance coverage, No. (%)**	**…**
Public^a^	47 (37.0)
Private^b^	51 (40.2)
Private via ADAP	25 (19.7)
Uninsured	4 (3.1)
**Had insurance change after initiation, No. (%)**	…
No	74 (58.3)
Yes	53 (41.7)
**Among patients with an insurance change (n = 53)**	
**No. of insurance changes, No. (%)**	…
1	32 (60.4)
2	11 (20.7)
3	7 (13.2)
4	3 (5.7)
**Caused delay in dosing or need for oral bridge, No. (%)**	…
Yes	6 (11.3)
No	47 (88.7)

Abbreviation: ADAP, AIDS Drug Assistance Program.

^a^Public: Medicare, Medicaid, Tricare.

^b^Private: via employer, Marketplace.

### Insurance Coverage and Delays

At the time of LA CAB/RPV initiation, 47 (37.0%) had public insurance (Medicare, Medicaid, Tricare), 51 (40.2%) private (via employer, Marketplace), 25 (19.7%) ADAP-sponsored private, and 4 (3.1%) were uninsured. Nearly half (53/127 or 41.7%) of patients had ≥1 insurance change after LA CAB/RPV initiation. Of the 53 patients with insurance changes during the study period, 32 (60.4%) had 1, 11 (20.7%) had 2, 7 (13.2%) had 3, and 3 (5.7%) had 4, totaling 87 distinct changes. Six patients experienced a delayed injection or need for an oral bridge as a direct consequence of an insurance change. One patient received an injection 1 day outside of their injection window, and the other 5 received appropriately timed oral bridging therapy lasting 3 weeks to 1 year, with no gaps in ART coverage. No virologic failures occurred in the cohort.

### Median Time to Coverage Change

The uninsured group had the least amount of time until an insurance change as compared with all other payor group types, with median days to change of 51 (uninsured) vs 300 (public; *P* = .009), 51 (uninsured) vs 378 (private; *P* = .002), and 51 (uninsured) vs 300 (private via ADAP; *P* = .023) (Table 2).

**Table 2.** Comparison of Median Days to Change Between Payors

	Insurance Coverage Before Change				
	Public (n = 57)	Private (n = 65)	Private via ADAP (n = 29)	Uninsured (n = 10)	*P* Value
Days until change	…	…	…	…	.0020^a^
Median (range)	300.00 (7.00–1102.00)	378.00 (4.00–1097.00)	278.00 (53.00–914.00)	51.00 (0.00–356.00)	

Abbreviation: ADAP, AIDS Drug Assistance Program.

^a^Kruskal-Wallis *P* value.

### Themes Underlying Insurance-Related Delays

Among the 6 patients with delays or use of an oral bridge, several themes emerged. First, loss of Medicaid or other insurance benefits, often unknowingly or unexpectedly, left patients not only vulnerable to delayed injections but also without a payor source to cover oral ART for bridging. Though ADAP programs and industry-sponsored patient assistance programs (PAPs) are potentially a valuable resource in bridging coverage, limited patient engagement in program enrollment processes, compounded by social barriers, hindered timely injection delivery. Second, patients covered by Medicaid who transferred care between states were at risk for treatment gaps, as both transfer of Medicaid coverage and new PA processes can take days to weeks. Additionally, in buy-and-bill scenarios, delays or denials of claim payment by insurers despite approved PAs left patients and providers hesitant to move forward with subsequent LAI treatment. Finally, patient self-enrollment in new out-of-network plans or plans with poor medication coverage resulted in lack of access to LAI therapy, difficulty obtaining oral ART for bridging, and, in some cases, transfer of care. The delays are described in detail in Supplementary Table 1.

### Mitigation Strategies and Postimplementation Outcomes

Following the first insurance-related delay in early 2023, we implemented multiple safeguards to help mitigate these problems in the future:

- Insurance verification at multiple time points:
  - Monthly verification at the beginning of the month in which the injection is due to allow time to secure medication/obtain PA if a change has occurred
  - Additional coverage re-verification the week before the scheduled injection, as some patients experience midmonth loss of insurance
- Bridging workflow should gaps in coverage still occur:
  - Formalized workflows with state ADAP programs to facilitate rapid coverage of medication during coverage transitions (oral bridge or injectable)
- Insurance check-in at each injection visit by experienced, in-house staff with counseling aimed at preventing and navigating coverage lapses to avoid treatment delays
- Regular screening for Ryan White eligibility and assistance with enrollment for qualified patients
- Familiarity with drug company PAPs and how to advocate for expedited PAs if needed
- Targeted education emphasizing the importance of notifying the care team about insurance changes
- A standardized oral bridging protocol to ensure uninterrupted treatment during unavoidable gaps in coverage
- Expedited and clear workflows for transfer patients already receiving LA CAB/RPV who are establishing care

After implementing these processes, no further insurance-related injection delays occurred after June 2024.

## DISCUSSION

Nearly half of patients receiving LA CAB/RPV at the UNMC SCC during the study period experienced 1 or more insurance changes. These changes may cause delayed injections or need for an oral bridge, which can lead to virologic failure. The most common causes included complete lapses in coverage quickly followed by another change when coverage is regained, delays in PA approval, and patient-initiated enrollment in out-of-network plans, which often resulted in unaffordable out-of-pocket costs.

Insurance-related disruptions were also observed during transitions of care, particularly when patients transferred into the clinic midcycle. The timing of transfer proved critical, underscoring the need for proactive coordination between sending and receiving sites.

Clinics implementing LA CAB/RPV must develop systems to proactively address insurance changes in a timely manner to avoid adverse patient outcomes. After implementing a structured set of mitigation strategies—including monthly insurance verification, formalized workflows for coverage gaps, rapid coordination with ADAP, targeted patient education, and standardized bridging protocols—we observed no insurance-related injection delays after June 2024 and no virologic failures throughout the study period. These outcomes highlight the importance of proactive, multidisciplinary systems for maintaining continuity of LAI ART.

This study has a few limitations. It is a single-center study in a Medicaid-expanded state with LA CAB/RPV on the ADAP formulary. Lack of Medicaid expansion and ADAP formulary variability would further affect access to LA CAB/RPV. Additionally, this site primarily utilizes a buy-and-bill model for medication procurement. Clinics relying on alternative methods of distribution such as specialty pharmacy dispensing may encounter different workflows and potential sources of delays, though this would not affect the number of insurance changes.

In summary, insurance changes are common in individuals receiving LA CAB/RPV, but their consequences can be mitigated through deliberate workflows and proactive monitoring. Clinics implementing LAI ART should develop systems to identify and address insurance changes in a timely manner to ensure uninterrupted treatment and optimal patient outcomes.

## Supplementary Material

ofag211_Supplementary_Data

## Supplementary Data

Supplementary materials are available at *Open Forum Infectious Diseases* online. Consisting of data provided by the authors to benefit the reader, the posted materials are not copyedited and are the sole responsibility of the authors, so questions or comments should be addressed to the corresponding author.
