# Supplementary material for: Real-World Impact of Insurance Changes on Long-acting Cabotegravir Plus Rilpivirine Delivery
Source: Open Forum Infect Dis. 2026 Apr 3;13(4):ofag211. doi: 10.1093/ofid/ofag211 (PMC13103877; doi:10.1093/ofid/ofag211)
Supplement: ofag211_Supplementary_Data [file ofag211_supplementary_data.docx]

**Supplemental Materials:**

| Table 1: The Six Delays in Detail | | |
| --- | --- | --- |
|  | **Problem** | **Outcome** |
| **Patient 1** | - Incidentally noted to have lost Medicaid after ER visit review, needed to enroll in ADAP to pay for medication which took about 1 month   *** because of this patient we started doing pre-verifications the month the patient’s injection is due | - Injection was given 1 day late - An oral bridge unable to be given for multiple reasons (financial, lack of housing, transportation, communication, etc.) |
| **Patient 2** | - Insurance denied payment on claims despite presence of prior authorization (PA) - Took several months to appeal and no one was comfortable proceeding without written confirmation that past and future doses would be covered | - Oral bridge for 7 months without gap in ART - Restarted injections once appeal was approved |
| **Patient 3** | - Transfer into our clinic on LA CAB/RPV with out of state Medicaid - Had to apply for in-state Medicaid (approved 3 days after patient initially contacted us) and get PA approved (3 weeks from PA submission to approval) | - Oral bridge for 3 weeks without gap in ART - Restarted injections once Medicaid approved |
| **Patient 4** | - Patient changed insurance and self-enrolled in out-of-network (OON) plan, discovered during pre-verification - Patient changed plan back to an in-network (IN) one as they did not want to transfer to an IN facility | - Oral bridge for 1 month without gap in ART - Restarted injections once insurance switched |
| **Patient 5** | - Self-enrolled in OON plan, discovered during pre-verification - Patient changed plan back to an IN one as they did not want to transfer to an IN facility | - Oral bridge for 1.5 months without gap in ART - Restarted injections once insurance switched |
| **Patient 6** | - Self-enrolled in an OON plan that also did not cover LA CAB/RPV - Transferred care temporarily until insurance was changed to an IN plan | - Oral bridge for twelve months without gap in ART - Restarted injections once insurance changed and re-established with our clinic |
